# Veterinary Medicines

**Published:** 1883-04

**Authors:** 


### REVIEWS.

VETERINARY MEDICINES—THEIR ACTIONS AND USES. By Finley Dun. Sixth Edition. Published by W. R. Jenkins, New York. 8vo., 676 pages.

The long and severe test of so large a number of editions, and especially where used as a text book in the various Veterinary Colleges of Europe and America, and the English Colonies, is a compliment not acquired without intrinsic worth.

This book is printed in large type, in the best style of modern bibliography. The arrangement into alphabetical order, according to the English nouns of veterinary medicines, is both convenient and admirable. Its entire devotion to Materia-Medica is another recommendation as a text book.

The scientific treatment of the numerous subjects considered is most admirable. There is no ambiguity in any part of the work. In fact Finley Dun's ability is conspicuous in every part. No matter what subject his pen touches it is treated in a creditable manner. The author is one of the ablest writers on the various subjects of Comparative Medicine.

